# Factors Associated with Fatal Occupational Accidents among Mexican Workers: A National Analysis

**DOI:** 10.1371/journal.pone.0121490

**Published:** 2015-03-19

**Authors:** Mery Gonzalez-Delgado, Héctor Gómez-Dantés, Julián Alfredo Fernández-Niño, Eduardo Robles, Víctor H. Borja, Miriam Aguilar

**Affiliations:** 1 Escuela de Salud Pública de México, Cuernavaca, México; 2 Centro de Investigación en Sistemas de Salud (CISS), Instituto Nacional de Salud Pública, Cuernavaca, México; 3 Centro de Información para Decisiones en Salud Pública (CENIDSP), Instituto Nacional de Salud Pública, Cuernavaca, México; 4 Coordinación de Salud en el Trabajo, Instituto Mexicano del Seguro Social (IMSS), México D.F., México; 5 Unidad de Atención Primaria en Salud, Instituto Mexicano del Seguro Social (IMSS), México D.F., México; University at Buffalo, UNITED STATES

## Abstract

**Objective:**

To identify the factors associated with fatal occupational injuries in Mexico in 2012 among workers affiliated with the Mexican Social Security Institute.

**Methods:**

Analysis of secondary data using information from the National Occupational Risk Information System, with the consequence of the occupational injury (fatal versus non-fatal) as the response variable. The analysis included 406,222 non-fatal and 1,140 fatal injuries from 2012. The factors associated with the lethality of the injury were identified using a logistic regression model with the Firth approach.

**Results:**

Being male (OR=5.86; CI95%: 4.22-8.14), age (OR=1.04; CI95%: 1.03-1.06), employed in the position for 1 to 10 years (versus less than 1 year) (OR=1.37; CI95%: 1.15-1.63), working as a facilities or machine operator or assembler (OR: 3.28; CI95%: 2.12- 5.07) and being a worker without qualifications (OR=1.96; CI95%: 1.18-3.24) (versus an office worker) were associated with fatality in the event of an injury. Additionally, companies classified as maximum risk (OR=1.90; CI 95%: 1.38-2.62), workplace conditions (OR=7.15; CI95%: 3.63-14.10) and factors related to the work environment (OR=9.18; CI95%:4.36-19.33) were identified as risk factors for fatality in the event of an occupational injury.

**Conclusions:**

Fatality in the event of an occupational injury is associated with factors related to sociodemographics (age, sex and occupation), the work environment and workplace conditions. Worker protection policies should be created for groups with a higher risk of fatal occupational injuries in Mexico.

## Introduction

The concept of “decent work” proposed by the International Labour Organization (ILO) to its member countries includes the promotion of the equality, safety and dignity of workers worldwide [1]. To this end, the measurement of sensitivity indicators—such as the number of occupational injuries, illnesses and deaths—provides a complete epidemiological panorama of the health and safety status of workers [1].

Nevertheless, the records used to determine advances in decent work generally are not sufficiently reliable. This is due to several factors, primarily: lack of social security coverage for workers in the informal sector [2], workers’ lack of knowledge about the risks to which they are exposed [3], underreporting in the formal sector, and lastly, inadequate registry and notification systems that do not permit comparisons among and within countries because of a lack of standardization among statistics [4–7].

The World Bank calculates that 60% of the population over 15 years of age is employed worldwide [8]. This is the population requiring resources and efforts to meet the goals for decent work, and which is potentially at risk of occupational injuries and illnesses. The number of occupational injuries and illnesses calculated by the ILO presents cause for concern, with 317 million occupational injuries resulting in an absence from work of at least three days, and 160 million cases of non-fatal occupational illnesses [6]. In addition, 6,300 workers die due to occupational causes daily, 5,500 of which are caused by occupational illnesses and 800 by occupational injuries, resulting in 2.3 million deaths annually—surpassing the deaths from AIDS (1.6 million) reported worldwide for the year 2012 [6,9,10].

Deaths due to occupational injuries and illnesses in all countries not only exceed deaths from AIDS and other public health diseases but also have high economic, social and family costs [11,12], equivalent to a 4% decrease in the Gross Internal Product (GIP) and 2.8 million dollars in direct and indirect costs worldwide [6].

Generally speaking, it is well-known that the economic costs of fatal and non-fatal injuries reflect negatively on businesses due to increased staff turnover resulting in more training needs, increased absenteeism, decreased production and higher insurance and workers compensation premiums [11,13–15]. In the case of the worker, when the injury is not fatal these costs are expressed in a loss of income and medical and rehabilitation expenses. Lastly, the cost to society involves losses in capital, higher health expenses, human deaths and reductions in the workforce [11,15–16].

In Mexico, the indicators of decent work show that 61.7% of the economically active population (EAP) is men, 13.3% of whom work in the primary sector, 24.4% in the secondary and 60.8% in the tertiary sector. In addition, only 35.6% have access to health institutions and 28.6% work in the informal sector [17]. With respect to fatal injuries, the Secretary of Labor and Social Welfare (Secretaría del Trabajo y Previsión Social; STPS, Spanish acronym) reported 1,152 deaths due to occupational risks, representing a rate of 0.74 per 10,000 workers at the national level for the year 2012 [18].

In addition, various studies have been performed in Mexico to evaluate several aspects related to this issue, including: risk factors associated with injuries in the construction sector [19]; consequences and problems related to the registry of occupational injuries occurring in the population without social security and treated in hospitals affiliated with the Mexico City Secretary of Health [20]; accumulated years of potential losses in productive life due to [21]; risk factors related to hand injuries in the beverage industry [22]; and the effect of underreporting of occupational accidents at the state and national levels [4][23]. Nevertheless, previous investigations have not addressed fatalities in the event of occupational injuries at the national level.

**Fig 1:**
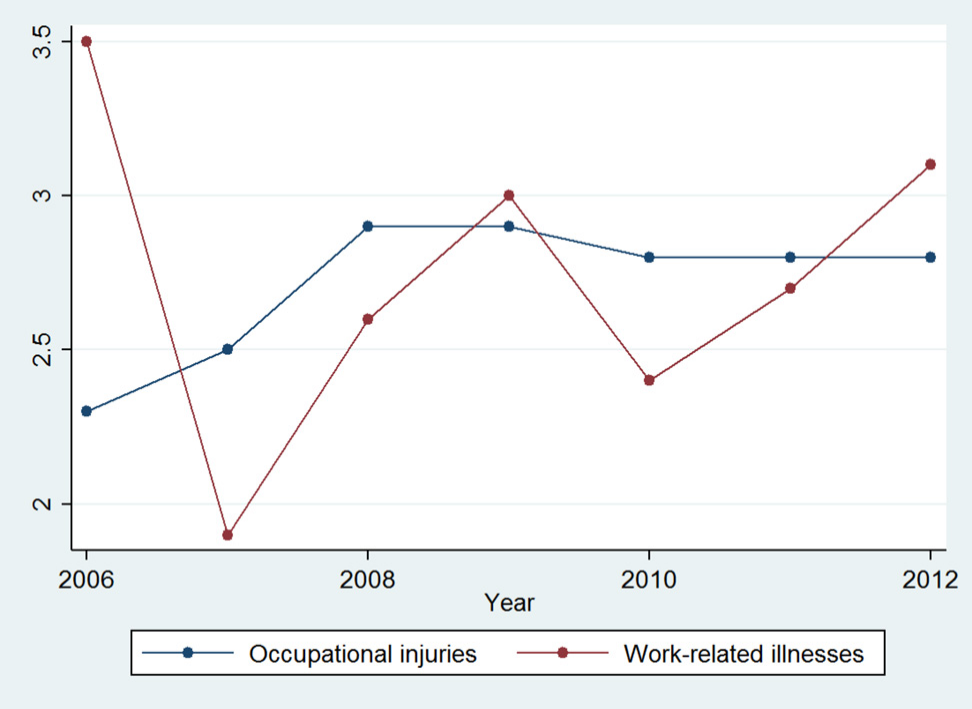
Rate of work-related illnesses and injuries per 100 employees, in the IMSS, 2006 to 2012.

The Mexican Institute of Social Security (Instituto Mexicano del Seguro Social; IMSS, Spanish acronym) is the only Mexican institution which has a national information system with good quality and coverage, about the occurrence of occupational injuries and their lethality in Mexico. In 2012, a total of 824,823 companies were affiliated with the IMSS, which had information for 15,671,553 workers with workers’ compensation insurance, 63.8% of whom were men. This institution represents the highest percentage of affiliated workers (together with their families) in the country’s formal sector (30.42% of the national population is affiliated with the IMSS). Given this level of national representativeness and coverage and its systematic collection of the information most relevant to each injury, the IMSS undoubtedly offers an ideal scenario to study occupational injuries in Mexico. Furthermore, according to its information system, an increase was observed in the occupational injury rate for this subpopulation, from 2.3 in 2006 to 2.8 per 100 workers in 2012 [24] (Fig. 1).

Several studies have associated the lethality of occupational injuries with factors pertaining to the worker—primarily sex, age, occupation, performing unsafe practices, length of employment and the degree of training [25]. Meanwhile, other studies have suggested that factors related to companies [26], especially organizational and normative aspects [27], could be even more relevant to the lethality of an injury than individual factors. The objective of this study is to identify the factors associated with fatality in the event of an occupational injury at the national level in Mexico using the Occupational Risk Information System database from the IMSS [28, 29]

## Methods

### Ethical Statement

The present analysis is based on secondary information from the IMSS information system. Written informed consent was given by participants (or their families in the case of the fatal events) for their records to be used in the information system. This consent was obtained at the time of the injury by the IMSS. For the present study, the records were used with the authorization of the IMSS and complete confidentiality was maintained for all of the analyses, including both individual and company records. To this end, the working database was anonymous and contact data were excluded. The research protocol was approved by the research ethics committee of the Mexican National Institute of Public Health (In Spanish: Instituto Nacional de Salud Pública).

#### Study Design and Sources of Information

A secondary analysis was performed based on information collected by the IMSS National Occupational Risk Information System. This system collects information from medical units, including reports related to occupational illnesses and injuries as well as deaths of those insured by IMSS throughout the entire Mexican republic.

This occupational injury database contains cases with no consequences as well as those resulting in temporary disability, partial and total disability, and death.

### Sample

The initial sample included 420,220 records related to fatal and non-fatal occupational injuries at the national level for the year 2012, of which 12,854 records were excluded due to the lack of information related to basic sociodemographic data (length of employment and occupation), company characteristics (sector) or type of injury (external cause, physical risk, unsafe act). In addition, 2 records were eliminated which were duplicate deaths and another 2 for which the consequence of the event could not be determined. For cases in which there was less than one week difference between the non-fatal and fatal injury for the same worker, the diagnostic of the external cause and the nature of the injury were considered suggestive of death due to the same injury and were therefore registered in the database as fatal (n = 5 records).

The sample also included 17,593 observations in which a worker experienced more than 1 non-fatal event (94.4% of which involved only 2 events). Considering all of the above, the final analytical sample included 407,362 records, 9,666 of which were occupational injuries without disability and without sequels, 396,556 were temporary disability without sequels, and 1,140 were deaths. Cases resulting in permanent disability were not included because of a lack of information, since it was not possible to obtain this database.

### Outcome Variables

#### Consequence of the occupational injury.

For the regression model, a dichotomous variable was generated by assigning a value of 0 to non-fatal injuries and 1 to fatal injuries resulting in the death of the worker.

We used the definitions established by the Mexican Law Labor [30]. Occupational Injury (more precisely named “work-related accident” in the Mexican Law) is defined as: “any immediate or posterior organic injury or functional distress, or any death, suddenly produced by performing a task at work, or as a result of work, regardless of the place or time of occurrence. Included in this definition are accidents produced as the worker travels directly from home to work, and vice versa” Free translation- [30]. An occupational injury is determined as follows. First, the potential accident is recorded in the system once the clinical opinion of the health practitioner is received by the occupational health team in each medical unit. Then, the accident is evaluated in the medical unit, using standardized questionnaires, the clinical history and documents from the company. Finally, the company could also be asked to report more information about the events that led to the accident. With this entire information, the decision is made as to whether to classify the accident as a “work-related accident”.

All the occupational injuries were treated at the IMSS, where a trained physician determined the existence of sequels or disability, as applicable, according to standardized clinical guidelines.

Fatal injuries were reported to the IMSS National Occupational Risk Information System. The occupational health physician in charge of each medical unit determined whether the cause of death was an occupational injury, based on the clinical history and other key documents.

### Explanatory Variables

The explanatory variables for fatality in the event of an injury are classified according to 3 groups:

Sociodemographic characteristics: sex, age (in years), length of time employed (<1 year, 1 to 10 years, 11 to 20 years and 21 years or more) and occupation of the worker at the time of the occupational injury (based on the International Standard Classification of Occupations (ISCO) by the International Labour Organization, for 88 occupations) [31].

Company characteristics: risk class, which determines the classification of the diverse economic activities and industrial areas, categorized from higher to lower worker exposure to risk (from the Catalogue of Activities, Title 8 of the Social Security Law pertaining to affiliation, classification of companies, tax collection and fiscal legislation) [32].

The economic activity was classified according to 7 categories: (1) agriculture, livestock, fishing, hunting; (2) mining; (3) manufacturing; (4) construction; (5) business; (6) transportation; and (7) services. These sectors correspond to the economic activities in which occupational injuries occurred and were adapted according to the classification of economic activities by the INEGI national jobs survey [33].

The destination region: corresponds to the location of the companies where the injuries occurred. These include: northwest (Baja California, Chihuahua, Baja California Sur), north (Aguascalientes, Coahuila, Durango, Nuevo León, San Luis Potosí, Sinaloa, Sonorora, Zacatecas), west (Jalisco, Michoacán, Nayarit, Colima), central (Mexico City, Puebla, Querétaro, Morelos, Tlaxcala, Mexico State, Guanajuato, Hidalgo), gulf (Tabasco, Tamaulipas, Veracruz), southern (Oaxaca, Chiapas, Guerrero) and the Yucatan Peninsula (Campeche, Quintana Roo and Yucatán).

### Characteristics related to the injury itself

Three variables are included: unsafe act, physical risk and external cause. In terms of physical risk (attributable cause), the identifiable cause in the investigation is the one considered by experts to be the most likely trigger of the injury (adverse workplace conditions, inadequate conditions related to work materials, hazardous contact with tools and energy, physical loads, deficient work organization and prevention management, and adverse factors related to the work environment (see Table 1 for detailed definitions). In terms of external causes, these were identified based on the wide-used ICD-10 codes. The variable “unsafe act” (whose categories are also described in Table 1) was included in the exploratory bivariate analysis but not in the final regression model, for reasons which will be explained later.

**Table 1:** Definitions of the categories of Unsafe act and Physical risk.

Categories	Definition
*Unsafe act:	Actions performed by the worker that involve the omission or violation of a work method or measure determined to be safe.
Adopt dangerous positions, attitudes and place, mix and combine in an unsafe manner	Includes unnecessarily adopting, placing or exposing to conditions, materials and elements in an unsafe manner.
Lack of attention to where one is stepping or one’s surroundings	Lack of attention to floors, stairs, public streets, slippery surfaces and scaffolds.
Failure to secure or prevent	Includes failure to stop or start vehicles, machinery or equipment. Failure to prevent the use of equipment in poor conditions and omit the use of warning signs.
Not using available personal protection equipment	Not using helmets, safety belts, aprons, goggles, gloves and masks available in the workplace.
Operating or working at an unsafe speed	Includes actions such as running in the workplace, jumping from heights, stocking or feeding too quickly and throwing materials or elements.
Inappropriate behavior at work	Includes abusing, frightening, distracting, bothering and clowning around.
No unsafe act	No unsafe act was reported for the injury.
Failure or unsafe act by third parties	Unsafe act not performed by the person suffering the injury.
Other unsafe acts	Includes use of unsafe personal attire and unsafe equipment and cleaning, lubricating, adjusting or repairing equipment in movement and/or with an electrical charge or pressurized load.
**Physical Risk**	**Cause attributed according to forensic investigation.**
Workplace conditions	Includes inadequate work spaces, inadequate control of transit through work areas, public hazards and inappropriate piling
Conditions of work materials	Includes equipment, machinery and materials that are broken, lacking shields, have no safety mechanisms or are defective or unsafe.
Tools	Worn, deteriorated cutting tools
Contact with energy	Includes conductors, connections and electrical switches that are not insulated, exposed or not grounded
Physical load	Includes the lack of support for lifting, moving and rolling loads.
Organization of the work and prevention management	Includes the use of hazardous methods, procedures and materials, lack of appropriate clothing for the work, unsuitable assignment of staff to perform specific activities
Factors related to the work environment	This category describes factors such as inadequate lighting, inappropriate pressure, excessive noise, inadequate ventilation and other factors in the work environment.
Not specified	Physical risk factors that are not specified or do not have sufficient information to be classified.

*For the logistic regression, “unsafe act” was considered as a dichotomous variable (Yes versus No), since the kind of unsafe act depends mainly on the specific occupation, and is highly variable in our sample.

### Statistical Analysis

Based on the bivariate analysis, the distribution of the quantitative variables was explored within each original outcome category (fatal versus non-fatal injury), and the Mann-Whitney U tests were used to determine whether the differences were statistically significant. For each qualitative variable, cross-tabulations were performed and evaluated with chi-squared tests and Cramér's phi for ordinal variables.

Finally, a logistic regression model was applied using the Firth approach with robust variance estimators for fatality as an outcome variable, and all the variables mentioned previously were included as independent variables, which were distributed according to the three groups mentioned (sociodemographics, company and injury). Firth approach was preferred because fatality is a very rare event in our data (0.28%) [34]. The correlation of the observations in the regression model was specified in order to account for the repeated measurements for individuals (more than one injury).

With regard to external cause, although this is closer related to the consequence of the event, it was included with the idea of adjusting the effect of the variables of interest by the clinical seriousness of the injury, based on the type of injury presented; that is, to evaluate whether the association of the explanatory variables was independent of clinical seriousness, which depends on many factors. Nevertheless, the results would not significantly change regardless of whether or not these two variables were included in the different previously adjusted models. As mentioned earlier, the regression model adjusted and presented here not only showed the best statistical performance but was also considered to be most consistent with our theoretical framework.

Pearson’s chi-squared tests and the Hosmer-Lemeshow test for goodness-of fit were used to evaluate the fit of the previously adjusted models. The performance of the different initial adjusted models was compared based on the Akaike Information Criterion (AIC) value and the best model was presented in our results according to these criteria [35].

For all the statistical analyses, tests were considered significant when *p*-values ≤0.01 and marginally significant when between 0.01 and 0.05. The *p*-values in the bivariate analysis were penalized using the classical Bonferroni correction. The analyses were performed with the Stata 12.1 statistical package (Stata Corporation, College Station, TX, USA).

## Results

Finally, this study analyzed 9,666 occupational injuries without disability, 396,556 with temporary disability and 1,140 fatal injuries.

First, for workers who had non-fatal injuries, the median value for days of disability was 24 (SD 5) and 98.6% of the injuries occurred during a continuous workday. The non-fatal injuries occurred primarily among workers with less than 1 year of employment (81.80%) and 31.29% occurred in the services sector. Another preliminary finding that is highly relevant to public health is that 0.54% (2,237) of the non-fatal occupational injuries occurred among children under 18 years of age, primarily in the services (825 injuries) and manufacturing (740 injuries) sectors. In terms of fatal injuries, workers who died from an occupational injury had, at most, one previous event during that year (1.75%); the total number of previous injuries could not be determined.

**Fig 2:**
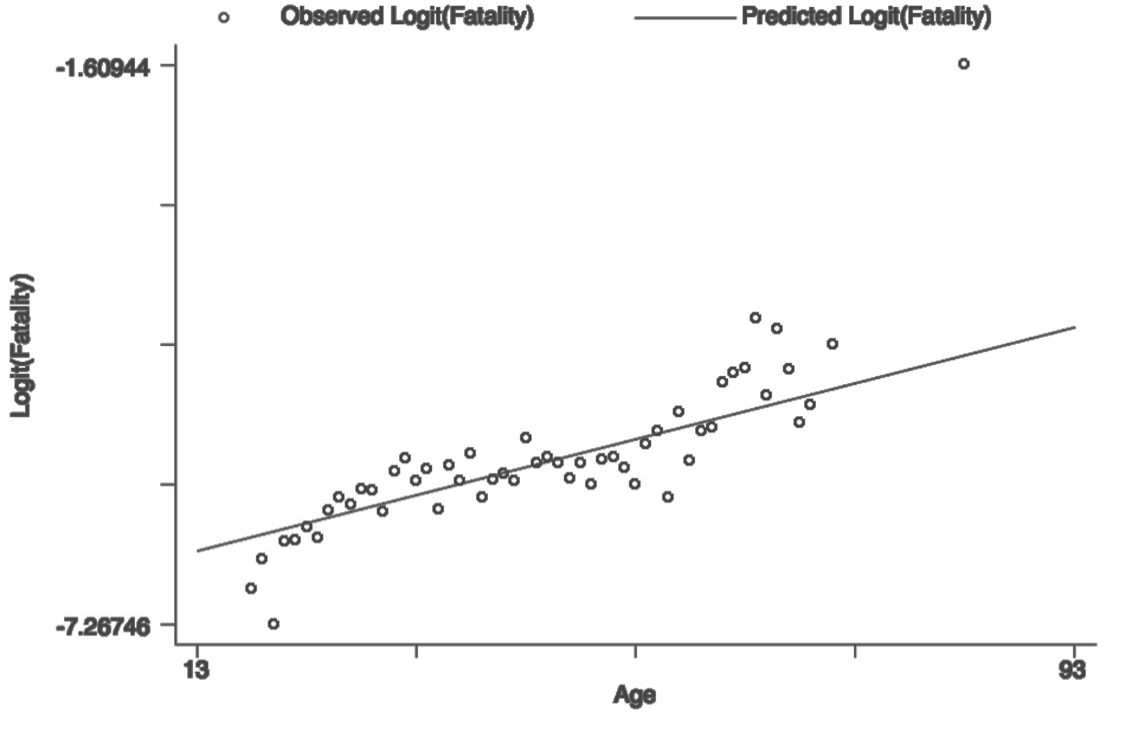
Relation between the logit of fatality versus continuous age.

Table 2 summarizes the primary characteristics of the study sample according to the final condition (fatal versus non-fatal injury). For each one, the *p*-values are presented from the bivariate tests performed to compare the distribution of each variable according to the condition. The median age of workers who died was 3 years older than those who had a disability and 4 years older than those who had a temporary disability without sequel (p<0.001). In fact, the relationship between the logit of fatality versus continuous age is practically linear, as observed in Fig. 2.

**Table 2:** Characteristics related to sociodemographics and the company, according to the type of consequence—non-fatal occupational injury (with temporary disability and without sequels, or without disability) and fatal occupational injury (death)—among workers affiliated with the IMSS in Mexico in 2012.

Variables	Non-fatal^+^ injury (n = 406,222)	Fatal^+^ injury (n = 1,140)	p value^++^
**Sex** (Male)	70.88	96.23	*
**Age** (years)	32 (25–42)	36 (29–45)	*
**Length of employment (Categorized)**			
Less than 1 year	81.80	77.07	*
1 to 10 years	10.14	14.59	
11 to 20 year	5.50	5.71	
21 years or more	2.55	2.64	
**Occupation**			
Members in positions of power, government and authorities	1.01	1.49	*
Scientific professionals and intellectuals	1.54	1.32	
Technicians and mid-level professionals	4.94	6.06	
Office workers	23.71	11.16	
Service workers and salespeople	15.70	10.72	
Farming and fishing	0.78	0.88	
Officials, operators and mechanical arts	11.84	17.57	
Machine operators and assemblers	14.87	29.53	
Workers without qualifications	25.58	21.18	
**Risk class of the company**			
Normal life risk	10.70	8.14	*
Low risk	30.76	15.13	
Medium risk	22.90	22.30	
High risk	14.44	13.54	
Maximum risk	21.21	40.88	
**Economic activity**			
Farming, livestock, fishing and hunting	2.36	4.48	*
Mining	0.93	3.58	
Manufacturing industry	24.99	11.47	
Construction	9.70	20.68	
Business	26.84	18,98	
Transportation	3.89	14.59	
Services	31.29	26.14	
**Destination region**			
Northwest	9.83	7.64	*
North	23,75	29,09	
West	15.53	13.27	
Central	36,86	32.60	
South	2.51	4.83	
Yucatan Peninsula	4.09	1.67	
**Unsafe act**			
Adopt dangerous positions, attitudes and place, mix and combine in an unsafe manner	14.15	8.08	*
Lack of attention to the to where one is stepping or one’s surroundings	24.31	19.86	
Failure to secure or prevent	37.98	28.38	
Not using available personal protection equipment	1.89	1.49	
Operating or working at an unsafe speed	0.68	2.02	
Inappropriate behavior at work	2.24	1.23	
No unsafe act	2.58	7.29	
Failure or unsafe act by third parties	6.76	26.10	
Other unsafe acts	9.40	5.54	
**Physical risk (attributable cause)**			
Workplace conditions	35.30	58.35	*
Conditions of work materials	11.09	8.35	
Tools	7.14	0.79	
Contact with energy	0.04	0.97	
Physical load	7.76	0.26	
Organization of the work and prevention management	29.65	24.25	
Factors related to the work environment	2.40	3.51	
Not specified	6.65	3.51	
**External cause**			
Transportation injuries	8.63	40.26	*
Falls	26.02	15.00	
Blows, crushing and traumatic contact	39.90	13.95	
Shooting and explosions	0.14	5.09	
Exposure to electrical current, radiation and temperature.	0.37	5.70	
Exposure to smoke, fire and flames	0.30	1.84	
Poisoning and exposure to toxic substances	0.13	0.70	
Various types of violence	2.92	12.37	
Other external causes	21.59	5.09	

+: Proportion(%) or median (Interquartile Range);

++: p-values adjusted by Bonferroni associated with chi-squared; Cramér's phi or Mann-Whitney U tests;

*p<0.001.

The proportion of men was significantly greater for deaths (96.23%) than for injuries resulting in temporary disability (71.01%) and injuries without disability (65.57%) (p<0.001). In fact, the male-female ratio was 25.5:1 for fatal injuries, while it was 2.4:1 for injuries with temporary disability and 1.9:1 for those without disability. Another noteworthy finding is that most of the injuries without disability (80.57%), with disability (81.83%) and resulting in deaths (77.07%) occurred during the first year of employment. Also notable is that most of the fatal injuries involved machine operators (29.53%) and most of the non-fatal injuries, with and without sequel (21.85% and 23.75%, respectively), involved office workers (*p*<0.001).

With respect to the employers’ characteristics, most of the non-fatal injuries occurred in low-risk companies (40.81 and 30.76% of non-fatal injuries with and without disability, respectively, versus 15.13% of fatal injuries; p<0.001), while most of the fatal injuries occurred in companies categorized as high risk (40.88%; p<0.001). In addition, with respect to the characteristics of the injury, the most frequent unsafe act for the three categories was the “failure to secure or prevent” (41.12% without disability, 37.90% with disability and 28.4% fatalities), which according to the context involves: starting or stopping plant vehicles or equipment without taking necessary precautions; failure to prevent the use of equipment in a poor state or out-of-service; not using locks; not balancing or securing oneself in the event of unexpected movements; not posting notices, signs or safety marks; and releasing or moving loads without giving adequate notice. It is worth highlighting that 26.1% of the fatal injuries were due to failures or unsafe acts by third parties, which was significantly higher than for injuries that were not fatal (p<0.001). Injuries that involved some type of transportation were proportionately higher for workers who died (40.3%) than those with temporary disability (8.7%) or without disability (4.6%) (*p*<0.001). Other characteristics related to injuries are presented in Table 2.

Table 3 presents the results from the logistic regression model for fatality, which should be interpreted as factors associated with death in the event of an occupational injury. In general, a statistically significant association was found for the following sociodemographic factors: being male (OR = 5.86; CI95%:4.22–8.14), continuous age (OR = 1.04; CI95%:1,03–1,06) and employed in the position for 1 to 10 years (versus less than 1 year) (OR = 1.37; CI95%:1.15–1.63).

**Table 3:** Logistic regression model for fatal occupational injuries (deaths) versus non-fatal occupational injuries (with temporary disability and without sequels, or without disability) among workers in the IMSS in 2012.

Variable	OR	CI 95%		p value
***Sociodemographic characteristics***				
**Sex** (Male)	5.86	4.22	8.14	*
**Age** (years)	1.04	1.03	1.06	*
**Length of employment** (Reference: < 1 year)				
1 to 10 years	1.37	1.15	1.63	*
11 to 20 year	1.08	0.83	1.4	n.s.
21 years or more	1.16	0.80	1.69	n.s.
**Occupation** (Reference: Office worker)				
Members in positions of power, government and authorities	1.71	0.92	3.16	n.s.
Scientific professionals and intellectuals	1.40	0.77	2.56	n.s.
Technicians and mid-level professionals	1.67	1.22	2.3	*
Service workers and salespeople	1.75	1.34	2.29	*
Farming and fishing	1.35	0.65	2.77	n.s.
Officials, operators and mechanical arts	2.71	1.86	3.95	*
Machine operators and assemblers	3.28	2.12	5.07	*
Workers without qualifications	1.96	1.18	3.24	*
***Company characteristics***				
**Risk class** (Reference: Less than the maximum)				
Maximum risk	1.90	1.38	2.62	*
**Economic activity** (Reference: Manufacturing industry)				
Farming, livestock, fishing and hunting	4.12	2.79	6.09	*
Mining	4.44	3.02	6.54	*
Construction	2.41	1.88	3.08	*
Business	1.49	1.16	1.92	*
Transportation	2.05	1.59	2.64	*
Services	1.76	1.37	2.27	*
***Injury conditions***				
**Physical risk** (Reference: tools)				
Workplace conditions	7.15	3.63	14.1	*
Conditions of work materials	5.36	2.66	10.81	*
Contact with energy	14.59	5.56	38.3	*
Physical load	0.49	0.13	1.86	n.s.
Organization of the work and prevention management	6.3	3.19	12.44	*
Factors related to the work environment	9.18	4.36	19.33	*
Not specified	3.88	1.86	8.12	*
**External cause** (Reference: blows, crushing and traumatic contacts)				
Transportation injuries	9.91	7.85	12.51	*
Falls	1.79	1.37	2.33	*
Shooting and explosions	60.51	43.17	84.81	*
Exposure to electrical current, radiation and temperature.	26.45	19.16	36.51	*
Exposure to smoke, fire and flames	12.58	7.68	20.6	*
Accidental poisoning and exposure to toxic substances	12.17	5.55	26.73	*
Various types of violence	11.5	9.08	14.56	*
Other external causes	0.85	0.62	1.17	n.s.
**Unsafe act**				
Yes (versus any kind of unsafe act)	1.48	0.85	2.58	n.s

OR = Odds ratio;

CI 95% = 95% Confidence Interval.

*p<0.01;

**p<0.05;

n.s: not significant.

With respect to occupation, and when compared to the possibilities of death for office workers, those with the greatest possibilities of death in the event of an injury were facilities and machine operators or assemblers (OR: 3.28; CI95%:2.12–5.07), mechanical arts workers (OR: 2.71; CI95%: 1.86–3.95), unspecified workers without qualifications (OR = 1.96; CI95%:1.18–3.24) and those working in services or business (OR = 1,75; CI95%:1.34–2.29).

With regard to the companies’ characteristics, greater possibilities of death in the event of an occupational injury were found only for those categorized as maximum risk, versus those with risks lower than the maximum (OR = 1,90; CI95%:1.39–2.62). In addition, the economic sectors most highly associated with fatality in the event of an injury were: livestock, agriculture, fishing and hunting (OR = 4.12, CI95%:2.79–6.09), mining (OR = 4.38, CI95%:3.00–6.39) and construction (OR = 2.41, CI95%:1.88–3.08). For all of the associations related to the economic sector of the company, the manufacturing industry was chosen as a reference since this sector is traditionally used as a comparison group, although the conclusions would be the same with a different reference group.

With respect to the conditions of the injury, those that particularly stand out as being associated with higher possibilities of fatality in the event of an occupational injury are: contact with energy (OR = 14.24; IC 95% 5.50–36.91), inadequate workplace conditions (OR = 7.15; CI95%:3.63–14.10), factors related to the work environment (OR = 9.18; CI95%:4.36–19.33), organization of the work (OR; 6.30, CI95%:3.19–12.44) and the condition of work materials (OR; 5.36, CI95%:2.66–10.81) (all of the above as compared to “tools” as the cause, which has the lowest risk)

An obvious limitation related to this variable (conditions of the injury) is that its categories are assumed to be mutually exclusive because of the way in which the data is collected by the information system. This is obviously not always the case. Nevertheless, this overlapping is certainly not as clear as the overlap that would be expected for the categories pertaining to “unsafe acts”. Actually, for this last variable, no statistically significant association is found and no other estimates of associations change. It therefore has no impact on the results obtained.

Lastly, the external causes associated with higher possibilities of death in the event of an occupational injury are presented at the end of Table 3, although given the main objectives of this analysis these were considered adjustment variables.

## Discussion

This study found statistically significant relationships between certain sociodemographic variables and the possibility of death in the event of an occupational injury. Some of these associations have been reported previously by other studies of the working population, such as: being male [4, 25, 36–46], age [4,36–40,47–51], length of time employed [12, 36,44,50] and occupation [19,20,50,52]. In addition, a greater possibility of a fatal outcome was found among workers in the construction, mining and industrial manufacturing sectors. These associations are consistent with other studies [12,53–59]. Agriculture/fishing [39,52,54,60], construction [19,20] and services [61] were also found to present higher possibilities.

Being male represented a higher risk of death in the event of an occupational injury, which could be explained by their occupations having a higher level of exposure to risks than women’s occupations [60–62]. Nevertheless, this analysis was adjusted for those variables. Another study in Mexico reaffirms what was found by the present work with respect to the work activities by males corresponding to higher risks [20].

The present study also found a 5% increase per year increase in age (OR 1.05; p<0.001) in the possibility of death in the event of an injury. While it has been reported that age groups from 25 to 44 years old have a higher proportion of fatal injuries [42,54,62–64], injuries among workers over 65 years of age are less frequent but apparently more often fatal [41,61,65]. The present study found a similar pattern, observing a frequency of 9.57% for non-fatal injuries in this age group and 15.73% for fatal injuries. This fact could be explained by decreased tolerance to injuries due to aging, comorbidities and processes pertaining to old age that increase the risk of death in the event of an injury [49,66].

In terms of the specific occupation, the present investigation found that workers without qualifications, facilities and machine operators and assemblers, workers in the services sector, salespeople in businesses and markets, and officials, operators and craftspeople in the mechanical arts present higher possibilities of death in the event of an occupational injury. In a study performed in the construction sector, the proportion of occupational injuries among manual laborers and bricklayers was consistent with the present study, since the manual workers also belong to the group of workers without qualifications [19–20]. This fact may be related to a lack of training [11, 64] for the tasks and a lack of access to safety measures on the job, and could also be explained by educational level [20,22, 55]. Unfortunately training and schooling variables were not available for the present study and, therefore, it was not possible to estimate their potential association.

In relation to the length of time employed in the position held when the occupational injury occurred, 1 to 10 years of employment was found to be associated with a higher possibility of death. For construction workers, the mean length of employment for occupational injuries was 6 years [12]. This could be explained by the perception of low risks given the routine nature, overconfidence and habits acquired with more time on the job, and would explain the differences found with less than one year of employment [67]. This particularly appears to be the case worldwide in sectors that are more hazardous [68]. In addition, after 10 years in a job, it is also possible that people protect themselves more from greater risks by taking more preventive measures, or because of their age their supervisors may assign them to tasks that have a relatively lower risk of a fatal outcome in the event of an injury.

In contrast, a study performed in the Autonomous Community of the country of Vasco, which was based on the injury communications system between companies and labor authorities, reported that workers with less than one month of experience presented 7.87% non-fatal injuries in 2007 versus 15.1% in 2011, whereas this remained stable for workers with over 20 years of experience [41]. Although this would likely be present in our data as well, it was not possible to determine the exact length of employment under 1 year because of failures in the system used to record this variable, which were identified during the course of this investigation.

An important finding by the present study was the significant association found between the unsafe act category called “failure or unsafe act by third parties” and fatality in the event of an occupational injury. Several investigations have reported human failure and unsafe acts as a determining factor for the presentation of fatal and non-fatal occupational injuries [22, 54]. In contrast, a study performed in Spain about workers’ risk perception regarding occupational injuries found that 25% of the injuries were due to overconfidence, habits, human error, carelessness, negligence and recklessness [69]. This fact can be explained by the perception workers have of the risks related to the activities performed in their work and how these activities become habits, which could change their perception of the reality of the tasks they perform [69].

Another aspect to be noted is the high percentage of physical risk in the presentation of fatal occupational injuries in Mexico in the categories related to the work environment (3.51%), workplace conditions (58.35%), work organization and prevention measures (24.25%). This finding is similar to that of a Spanish study which reported that the organization of the work and prevention measures were accountable for the largest proportion of the factors involved in fatal injuries among the working population in Spain [62]. Other studies have found the environment and workplace conditions to be relevant factors for causing fatal and non-fatal occupational injuries [46,59,69].

The findings by the present work can be understood in the context of theories about the causality of occupational injuries. These theories determine unsafe acts and unsafe conditions to be the immediate causes of occupational injuries, implicating factors related to the workers themselves and their behaviors as well as to the environment and workplace [67], as was also reported by the present study. In particular, our findings make it possible to recognize the fundamental importance of a company’s structural and organizational factors, which have at least the same or more weight than working individuals. Our findings also enable determining the groups most vulnerable to death in the event of an injury, such that they can serve as a basis for the development of protection strategies for the most vulnerable groups. New prospective studies which include the role of the most proximal causes as such as unsafe act by specific occupation, using models as pathway analysis, could complete our results.

This study has several limitations. One is not having measurements of certain variables reported by other studies worldwide, such as the level of schooling of the worker, socioeconomic level, training for the tasks to be performed by the worker, existence or lack of ongoing training programs and preventive maintenance programs for machines and equipment [19,20, 36, 54, 61]. In addition, the variables related to the condition of the injury should be carefully considered since they are prone to differential information bias given that it is not possible to dismiss (and it cannot be evaluated with the available information) the possibility of more rigorously and detailed investigations of the circumstances involved in cases of fatal accidents. This would tend to overestimate the strength of the association of these factors. Therefore, in the context of an information system, the evaluators of the causes of the injury unfortunately cannot be blinded to the outcome (fatal or non-fatal) and, thus, in this study the determination of the conditions of the injury would be differential (related to the information gatherers’ knowledge of the outcome and the hypothesis, potentially producing an evaluator bias). Nonetheless, a favorable aspect is that these procedures are performed according to standard protocols by specialized experts given the legal medical implications of each injury, fatal as well as non-fatal.

Another relevant limitation is not including as predictors of fatality the variables that measure the quality, time and overall conditions of the medical care provided for the injury. Therefore, the findings do not take into account differences in medical care among occupations, which may certainly occur even though all were insured by IMSS; although having the same insurer would undoubtedly minimize this problem to a certain degree. Consequently, the results of this work cannot be interpreted as causal since the causes of fatality in the event of an occupational injury are complex and this study does not include all of the relevant variables.

Another important limitation related to the scope of this type of study is that it does not include records of the working population in the informal sector and, therefore, cannot be extrapolated to this population [70]. Nevertheless, suggestive associations can be established given that the data has broad coverage and national representativeness, making it possible to identify the most vulnerable groups and potential determining factors. The limitations mentioned could be addressed with better data and the availability of the variables mentioned, and particularly with prospective follow-up, in addition to the development of methods (for example, structural equations analysis) that would make it possible to model the causal chain of a fatal injury. All the above-mentioned limitations are only a reflection of the fact that the information system was developed for public health and monitoring purposes rather than for epidemiological causal investigations.

On the other hand, no original epidemiological study would have the national coverage and representativeness provided by a good information system. Thus its limitations are the cost of the large advantages it provides. In addition, this preliminary study has actually served as feedback for the information system, improving the quality of its data. This will enable improving future research that can be conducted based on this system.

Furthermore, this investigation opens the door to propose new studies that would include the variables mentioned above in order to identify factors associated with fatality in the event of occupational injuries for each economic activity. It also opens the way to cohort studies to identify the psychosocial and risk perception factors involved in the presentation of fatal and non-fatal injuries, in order to design work-related health policies that promote the care and protection of workers. It would also be important to explore interactions with ethnicity, socioeconomic stratum, migration, labor conditions, employment and work stability, factors reported by studies in other countries as being related to the presentation of fatal and non-fatal injuries [27, 62, 68, 71].

## Conclusions

In conclusion, the results of this study suggest that sociodemographic factors (sex, age, length of employment and occupation) and those related to workplace conditions, the environment, the organization of the work and economic activities involving mining, agriculture, fishing, hunting and livestock are associated with the possibility of death in the event of an occupational injury. Companies are mostly responsible for these factors. This finding indicates that most deaths occurring in the event of occupational injuries can be prevented through the design of safety measures, hazard elimination or substitution, techniques and measures related to the organization of the work and by the implementation of risk management to protect workers in the workplace [27, 72].

In addition, the design and implementation of a national Occupational Injury and Illness Surveillance System is needed to identify the health conditions of this population. Furthermore, more rigorous legal control measures and occupational injury prevention programs need to be implemented by national and international institutions responsible for the monitoring and control of the health of workers in order to promote decent and safe work throughout the nation.
